# A119 ACUTE DIVERTICULITIS & CONCURRENT DIVERTICULAR BLEED: A RARE CLINICAL ENTITY

**DOI:** 10.1093/jcag/gwab049.118

**Published:** 2022-02-21

**Authors:** S Hasan, A Ilnyckyj

**Affiliations:** University of Manitoba Max Rady College of Medicine, Winnipeg, MB, Canada

## Abstract

**Aims:**

Colonic diverticula are mostly asymptomatic incidental findings on cross sectional imaging or colonoscopy. In the setting of known diverticulosis, complications like diverticulitis and diverticular bleeding occur in only 4% and 1% of patients respectively. It is unusual to see both complications expressed in the same patient and moreover, rare to see them expressed concurrently. We report a case of concurrent diverticulitis with diverticular bleed and expand on the clinical course.

**Methods:**

Case report

**Results:**

A 73-year old was diagnosed with sigmoid diverticulitis based on her clinical presentation and an abdominal CT scan. She was discharged home on oral antibiotics but presented a week later with painless rectal bleeding. In view of the ongoing diverticulitis, a colonoscopy was not pursued although it is the typical procedure of choice to manage active gastrointestinal bleeding. A CT scan was recommended as the alternate initial investigation, which revealed active colonic bleeding in the region of the hepatic flexure immediately adjacent to a diverticulum. A subsequent CT angiogram identified the middle colic artery to be the responsible blood supply to the region and embolization coils were deployed within this vessel in an attempt to achieve hemostasis.

Despite vascular intervention the patient continued to bleed and resuscitation with blood products was insufficient. A repeat CT scan ruled out any areas of colonic ischemia as well as any further active areas of extravasation. A decision was made to proceed with a total colectomy to provide definitive treatment of the diverticular disease and manage the bleeding. An urgent intraoperative colonoscopy was attempted but quickly abandoned due to suboptimal preparation and ongoing bleeding obscuring the view. The patient underwent a total colectomy with ileorectal anastomosis. On pathology there was extensive diverticulosis of the ascending and the sigmoid colon with diverticulitis and diverticular abscess cavities within the sigmoid colon.

**Conclusions:**

Diverticulitis and diverticular bleed are thought to be unrelated complications of diverticulosis involving distinct physiologic pathways (Figure 1). Although diverticular bleed may occur in patients with a history of prior diverticulitis, concurrent presentation of these two entities is extremely rare. In addition, diverticular bleed is mostly self-limiting. While endoscopic and vascular embolization are established treatment options, colectomy is rare for the management of diverticular bleeding.

We report a rare case of concurrent diverticulitis and lower GI bleeding. The presence of the sigmoid diverticulitis made interventional radiology a relatively safer option over endoscopy, which is the typical first line diagnostic and interventional procedure. Ongoing bleeding led to total colectomy and provided the patient with a definitive cure for her diverticular disease.

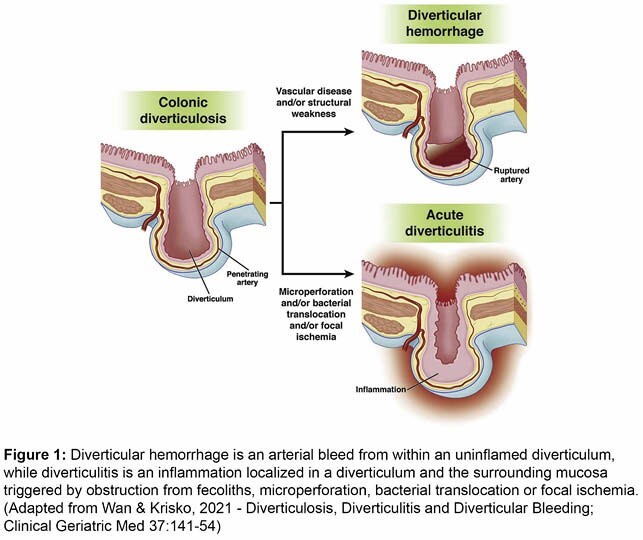

**Funding Agencies:**

None

